# Excimer-ultraviolet-lamp-assisted selective etching of single-layer graphene and its application in edge-contact devices

**DOI:** 10.1186/s40580-024-00442-5

**Published:** 2024-08-22

**Authors:** Minjeong Shin, Jin Hong Kim, Jin-Yong Ko, Mohd Musaib Haidari, Dong Jin Jang, Kihyun Lee, Kwanpyo Kim, Hakseong Kim, Bae Ho Park, Jin Sik Choi

**Affiliations:** 1 Department of Physics, Division of Quantum Phases and Devices, Konkuk University, 120 Neungdong-ro, Gwangjin-gu, Seoul, 05029 Republic of Korea; 2 Department of Physics, Yonsei University, 50 Yonsei-ro, Seodaemun-gu, Seoul, 03722 Republic of Korea; 3 Korea Research Institute of Standards and Science (KRISS), 267 Gajeong-ro, Yuseong-gu, Daejeon, 34113 South Korea

**Keywords:** Selective etching, Photochemical etching, Excimer UV, Single-layer graphene, Edge contact

## Abstract

Since the discovery of graphene and its remarkable properties, researchers have actively explored advanced graphene-patterning technologies. While the etching process is pivotal in shaping graphene channels, existing etching techniques have limitations such as low speed, high cost, residue contamination, and rough edges. Therefore, the development of facile and efficient etching methods is necessary. This study entailed the development of a novel technique for patterning graphene through dry etching, utilizing selective photochemical reactions precisely targeted at single-layer graphene (SLG) surfaces. This process is facilitated by an excimer ultraviolet lamp emitting light at a wavelength of 172 nm. The effectiveness of this technique in selectively removing SLG over large areas, leaving the few-layer graphene intact and clean, was confirmed by various spectroscopic analyses. Furthermore, we explored the application of this technique to device fabrication, revealing its potential to enhance the electrical properties of SLG-based devices. One-dimensional (1D) edge contacts fabricated using this method not only exhibited enhanced electrical transport characteristics compared to two-dimensional contact devices but also demonstrated enhanced efficiency in fabricating conventional 1D-contacted devices. This study addresses the demand for advanced technologies suitable for next-generation graphene devices, providing a promising and versatile graphene-patterning approach with broad applicability and high efficiency.

**Supplementary Information:**

The online version contains supplementary material available at 10.1186/s40580-024-00442-5.

## Graphical Abstract

This study introduces a novel selective etching method for single-layer graphene (SLG) using excimer UV irradiation under ambient conditions. Its high selectivity enables the precise removal of SLG, while leaving other graphene-based structures intact. Using this technique, we effectively fabricated edge-contact devices via SLG patterning with hBN masks. This advancement holds significant promise for expanding the applicability of graphene and paving the way for large-area applications.

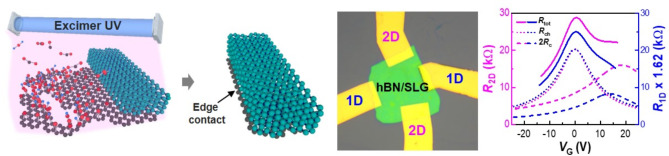

## Introduction

Graphene, comprising a single layer of carbon atoms arranged in a hexagonal lattice, is a two-dimensional (2D) material exhibiting unique properties owing to its layered structure and van der Waals interactions. These interlayer interactions contribute to various material properties that are influenced by both the intrinsic band structure of a single layer and the number of layers [1–3]. Owing to its extraordinary band structure, electrical conductivity, carrier mobility, and mechanical and optical properties, graphene has attracted significant attention from the scientific research community and the electronics industry. Precise etching techniques for fabricating novel graphene-based devices are necessary, particularly for the fabrication of one-dimensional (1D) edge contacts to reduce the contact resistance (*R*_*C*_) between 2D graphene channels and three-dimensional metal electrodes. This necessity stems from the progress in large-area, high-quality graphene growth technology through chemical vapor deposition (CVD), aiming at promoting industrialization and broadening graphene applications.

However, conventional etching methods for 2D materials encounter engineering constraints associated with controlling the edge state and optimizing experimental conditions. Various etching methods have limitations in terms of coverage, processing speed, cost, reproducibility for large-area processing, and excessive damage or contamination. Owing to these limitations, edge control in graphene-based electronic devices becomes challenging. Wet etching methods offer mild chemical reactions; however, wrinkles or irregularities may develop in graphene nanosheets during the solution process. Dry etching techniques, commonly used for patterning 2D materials by irradiating plasma of reactive gases, facilitate delicate patterning via electron beam lithography. However, oxygen molecule (O_2_) plasma etching, a conventional method for etching various 2D materials, induces charging damage and contamination that degrades their electrical properties. In contrast, hydrogen plasma etching enables selective etching at the edges over the basal plane of graphene but suffers from extremely slow etching rates, measured in sub-nanometers per minute. Therefore, using controlled O_2_ plasma etching for forming edge contacts for graphene in a vertical heterostructure with hBN requires a complex process. This process demands precise adherence to various experimental conditions, such as high vacuum, gas flow, voltage, and treatment time [4–6]. 

In this study, we propose a selective single-layer graphene (SLG) etching method as a patterning technique for 2D heterostructure devices, enabling efficient and uniform processing over large areas using an excimer ultraviolet (UV) lamp. Based on photochemical oxidation, our method exclusively targets the excimer UV-exposed SLG surfaces, offering easy control and clean processing. Our facile method etches SLG using excimer UV irradiation with a single wavelength of *λ* = 172 nm under ambient conditions for a sufficient duration of 240 s. The energy from the UV light selectively interacts with the basal plane of the SLG lattice by inducing the adsorption of oxygen groups on its surface. We demonstrate the selectivity of SLG etching and confirm that few-layer graphene remains undamaged through measurements using atomic force microscopy (AFM), Raman spectroscopy, and transmission electron microscopy (TEM). Moreover, we successfully fabricate hBN/SLG field-effect transistors (FETs) with 1D edge contacts using the UV-assisted etching method, highlighting its potential for integration in SLG-based electronic devices with low contact resistances.

## Results and discussion

**Fig. 1:**
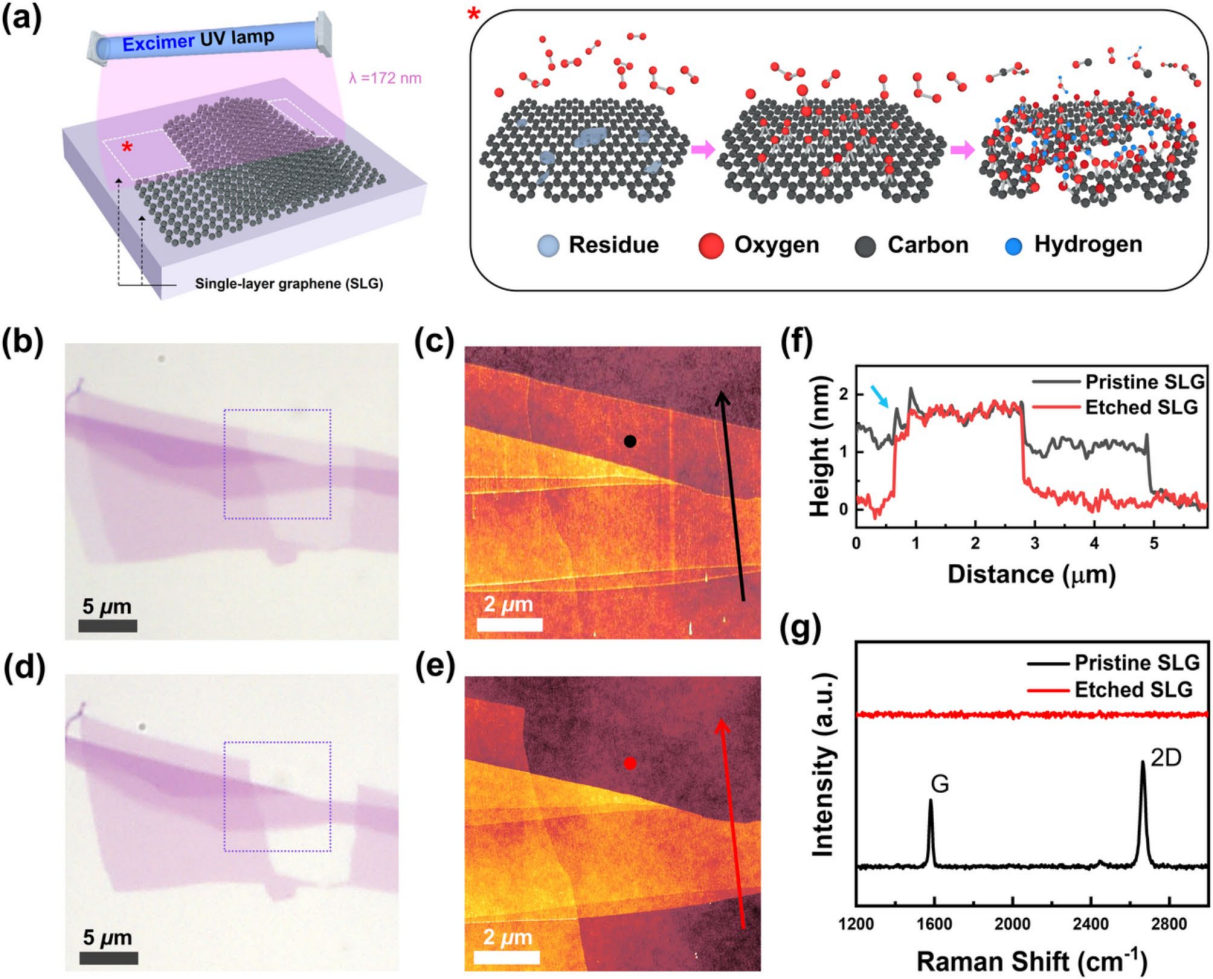
Selective etching of SLG among mixed layers of graphene. (**a**) Schematic of the experimental setup (left) and the photochemical etching process of SLG via excimer UV irradiation (right; red asterisk). Optical microscopic and AFM topographic images of pristine exfoliated graphene (**b**, **c**) before and (**d**, **e**) after 240 s of excimer UV irradiation. Dotted squares in (**b**) and (**d**) indicate the AFM scan area of (**c**) and (**e**). (**f**) Height profile comparison before and after excimer UV irradiation, measured along the black and red arrow lines shown in (**c**) and (**e**). (**g**) Changes in the Raman spectrum of SLG after 240 s of excimer UV irradiation. Raman spectra in (**g**) were obtained at the positions designated as black and red dots in the AFM topographic images (**c**) and (**e**)

Figure 1 depicts the experimental setup, process, and evidence of the selective etching of SLG. To observe the selectivity of the process, a mechanically exfoliated graphene sample containing various numbers of layers was irradiated with Xe_2_ excimer UV light of wavelength 172 nm at an illumination power density of 11.2 mW/cm^2^ for 240 s under ambient conditions. Compared to conventional UV etching methods, our excimer UV technique operates under simpler and easier processing conditions and demonstrates better performance with highly uniform and selective etching (Table S1 in SI). Figure 1a illustrates the experimental setup and the SLG etching process during excimer UV irradiation. Excimer UV energy generates ozone (O_3_), which produces O_2_ molecules and singlet atomic oxygen (O). Singlet atomic oxygen, which possesses a strong oxidation power, reacts with the SLG surface, gently removes organic residue contaminants, and generates volatile byproducts, such as CO_2_, H_2_O, and O_2_. (see Figure S1 in Supporting Information (SI)) Epoxy groups (C-O-C) form on the SLG lattice during oxidation, saturating at a 22% oxygen/carbon (O/C) atomic ratio. After reaching a saturated oxidation state, carbon–carbon covalent bonds break, leading to their combination with higher molecular weight functional groups such as –COOH. This phenomenon results in the formation of carbon vacancies in the basal plane of graphene, which subsequently grow into pores, initiating the etching of SLG (see Figure S2 in SI). Figure 1b-g shows the results of various measurements, confirming that the excimer UV treatment selectively removed only SLG among the different graphene layers. Lee et al. investigated this mechanism using a synthetic quartz UV lamp [7, 8], with a focus on the van der Waals-mediated interlayer interactions impeding the deformation of the top layer, which is crucial for attaching an oxygen group to the graphene lattice. The high kinetic barriers associated with the rearrangement of the carbon lattice hinder the covalent chemistry of the basal plane of graphene. A comparison of the optical microscopic (Fig. 1b and d) and AFM topographic (Fig. 1c and e) images confirms that only the SLG area was removed without leaving any trace, even at the atomic scale.

### Selective etching of SLG

Figure 1f and g further confirm the effectiveness and advantages of the proposed selective etching method for SLG. In the AFM line profiles (Fig. 1f), only SLG was removed, indicating a sharp height change at the edge of the etched graphene. This implies that there was no physical damage to the remaining graphene edges. Interestingly, the edges of pristine graphene sometimes exhibited spiked-up features (blue arrow in Fig. 1f), whereas the edges appeared to be stabilized after excimer UV irradiation. This phenomenon can be attributed to organic contamination at the cut edges of pristine graphene, which contain chemically unstable dangling bonds from the mechanical exfoliation process. The AFM cantilever tip may adhere to the edges during scanning, a phenomenon that disappears after excimer UV irradiation because of the cleaning effect. This distinguishes the excimer UV etching technique from conventional etching methods in terms of the atomically controlled termination, suggesting that our method enables the precise control and preservation of edges in the remaining graphene nanosheets.

Raman spectroscopy—commonly used in graphene research—provides extensive information on the structural stability, number of layers, doping states, and electrical properties of graphene [9–13]. In Fig. 1g, the Raman spectrum of the thinnest layer shows distinct SLG characteristics, with single Lorentzian G and 2D peaks at approximately 1580 and 2680 cm^-1^, respectively. These characteristic features disappeared completely after 240 s of excimer UV irradiation, and the Raman spectrum exhibited no residual D peak at ∼ 1350 cm^-1^ or a broad peak from amorphous carbon near the G peak position. This selective etching effect was confirmed by analyzing several exfoliated samples (Figure S3 in SI).

### Raman analysis of excimer UV-exposed multilayered graphene

**Fig. 2:**
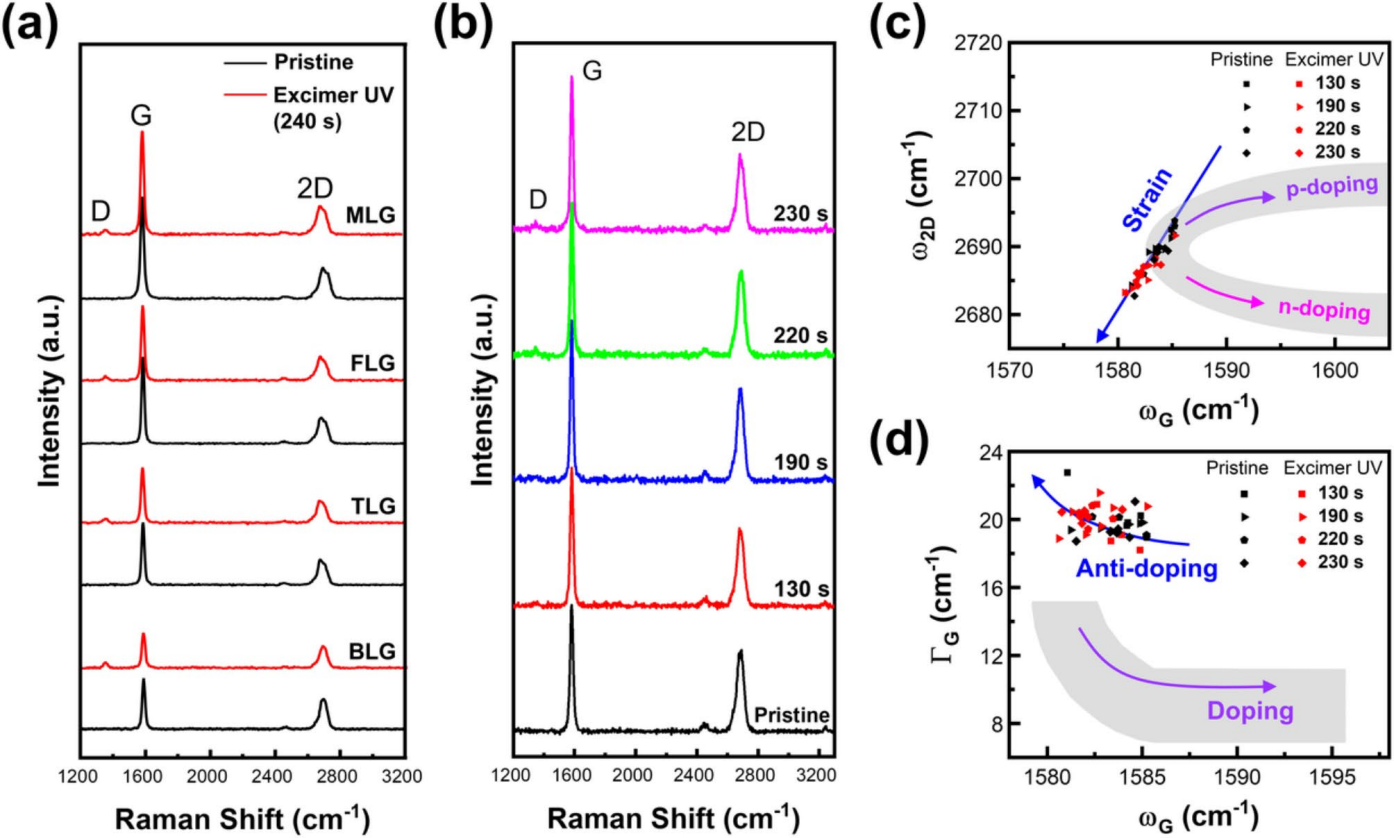
Raman spectrum analysis on MLG after 240 s of excimer UV irradiation. (**a**) Raman spectra of MLG with various layer numbers exposed to excimer UV light under etching conditions (240 s). The spectra of pristine and excimer UV-exposed graphene samples are represented by black and red lines, respectively. Distinct regions within a single exfoliated sample correspond to BLG, TLG, FLG, and MLG graphene. These layers are identified based on optical colors, Raman spectrum features, and AFM topographic heights. (**b**) Raman spectrum changes in BLG as a function of excimer UV irradiation time. (**c**) Strain and doping analysis of BLG based on the correlation between the G and 2D peak positions (ω), extracted from the data presented in (**b**). The blue arrow represents the reference line for biaxially strained BLG, as reported by Zabel et al. [14]. The colored curved arrows in the shaded area represent an experimental reference for doping effects observed by Fates et al. [15]. (**d**) Doping analysis of BLG by examining the relationship between the peak position and full width at half maximum (FWHM; Γ) for the G peak. The purple curved arrow with a shaded area shows the reference to the doping effect in BLG, as reported by Das et al. [16]. Each Raman spectrum was normalized using the Si peak intensity to 1, with the aligned position at 520 cm^-1^

To ensure the selectivity of the excimer UV etching process for a specific number of graphene layers, the complete removal of a particular layer is imperative. Similarly, it is important to verify that the etching process does not adversely affect the physical properties of the remaining graphene with different numbers of layers. Although we assessed the integrity of the remaining layers through optical microscopy and AFM topography, it was essential to demonstrate that they maintained their original structure and physical properties after the surface treatment process. Raman spectroscopy is the most effective method for confirming the physical properties of graphene, including its structural and electrical stabilities. Figure 2a shows the Raman spectral changes in the regions corresponding to bilayer (BLG), trilayer (TLG), few-layer (FLG), and multilayer (MLG) graphene within a single exfoliated graphene sample. The distinct Raman spectrum characteristics of each layer number remained consistent, except for a slight increase in the D peak (∼ 1350 cm^*−* 1^) after excimer UV irradiation, which is a layer-number-independent feature. In particular, when the Si peak intensity (*I*_Si_) value of the Raman spectrum is normalized to 1, the D peak intensity (*I*_D_) exhibited a uniform value of 0.018 *±* 0.004, while the G peak intensity (*I*_G_) increased with the number of layers from 0.12 (BLG) to 0.35 (MLG). Considering the significant chemical changes occurring simultaneously on SLG surfaces, it is difficult to associate the increase in the *I*_G_ with substantial chemical bonding, which would cause structural changes and electrical property degradation. Therefore, if the change in the D peak is insignificant, it may be considered a secondary effect arising from changes in strain and doping.

Figure 2b shows the excimer UV irradiation time-dependent Raman spectral changes in the BLG samples. The BLG samples were subjected to excimer UV irradiation for durations ranging from 130 to 230 s at arbitrary intervals (see Figure S4 in SI). Figure 2c exhibits the relationship between the 2D peak and G peak shifts (ω) for strain and doping analysis [15–17]. The ω_G_–ω_2D_ graph exhibits strain sensitivity under uniaxial or biaxial stress and is further influenced by extra charges, which impacts bonding lengths and non-adiabatic electron–phonon coupling. The blue arrow represents the reference line for biaxially strained BLG measured by Zabel et al. [14]. In addition, the purple and magenta curved arrows with shaded areas respectively represent the experimental references for the p- and n-doping effects observed by Fates et al. using gate voltage control in AB-stacked CVD graphene [15]. Variations in the ω_G_–ω_2D_ graph of the BLG fabricated in this study revealed a subtle increase in strain, which was further supported by statistical changes in ω_G_ (-1.63 cm^-1^) and ω_2D_ (-3.89 cm^-1^) before and after the excimer UV treatment. These changes were observed without a distinct dependency on irradiation time, even within the extended irradiation range of 130–240 s. This minor increase in strain aligns well with the reference line for the strained graphene after excimer UV irradiation, exhibiting no noticeable changes in doping. Figure 2d shows the correlation between the FWHM of the G peak (Γ_G_) and ω_G_, which facilitated the independent analysis of the doping effects. In this analysis, we referred to the doping effect observed by Das et al. in exfoliated BLG with gate voltage control [16]. Interestingly, only minor statistical changes in ω_G_ (-1.35 cm^-1^) and Γ_G_ (0.19 cm^-1^) were observed before and after excimer UV irradiation, despite a decrease in the doping level in our BLG. Considering that mechanically exfoliated graphene exhibits compressive strains and p-doped states, MLG appears to maintain its physicochemical properties without being significantly affected by the excimer UV exposure.

### TEM analysis of selective SLG etching on hBN

**Fig. 3:**
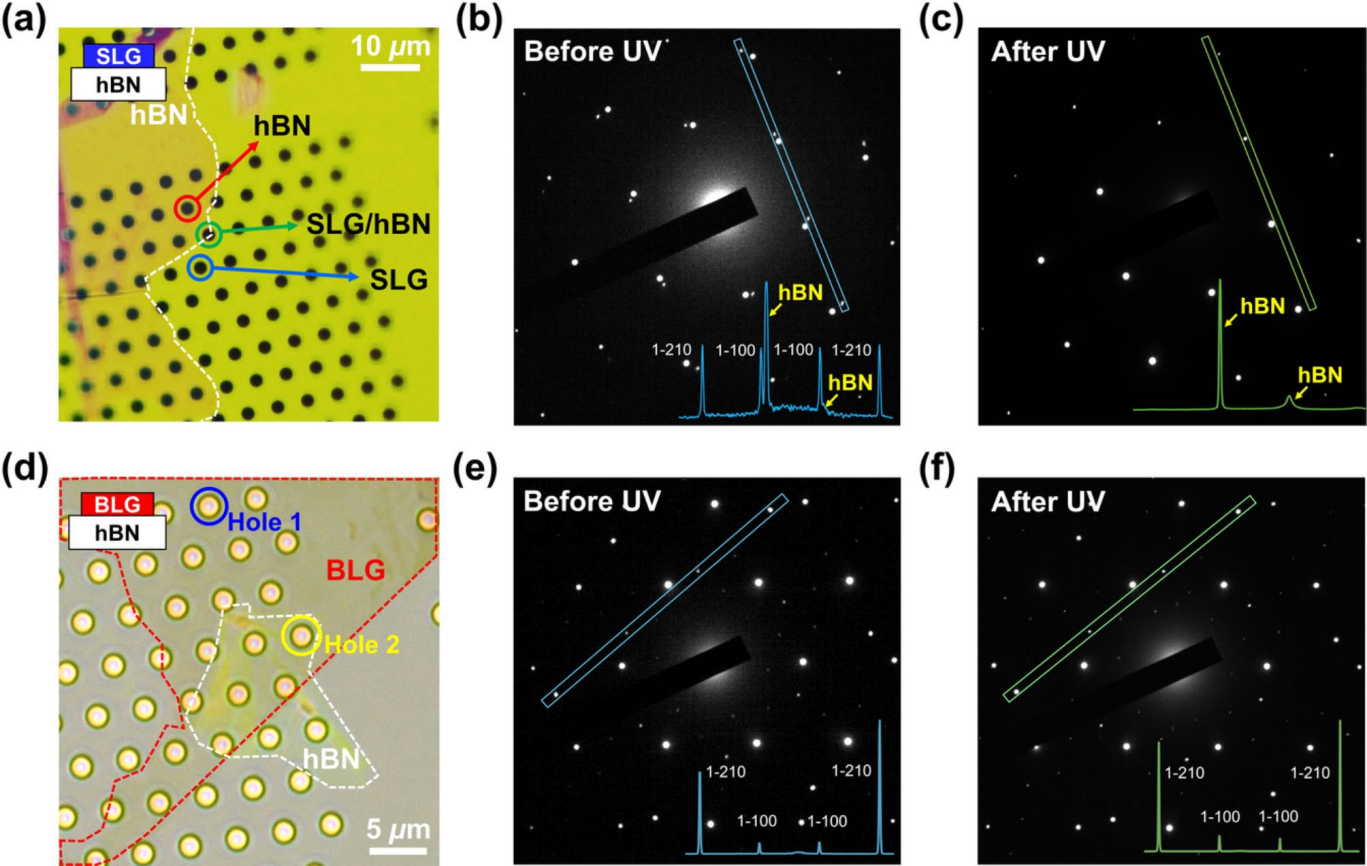
TEM characterizations of graphene on hBN after 240 s of excimer UV irradiation. (**a**) TEM sample preparation consisting of SLG, hBN, and the TEM grid. The inset illustrates the structural order of graphene/hBN on the TEM grid. (**b**, **c**) Representative SAED patterns and corresponding intensity profiles (inset) along designated lines obtained at SLG/hBN before (**b**) and after (**c**) excimer UV etching. (**d-f**) Same dataset as (**a-c**) for BLG/hBN. Hole 1 and Hole 2 indicate BLG-only and BLG/hBN structures, respectively. Representative SAED patterns in (**e**) and (**f**) were obtained at Hole 2

Direct observation of the atomic periodic structure and number of layers was achieved through TEM, with the sample prepared by transferring it onto a TEM grid, as shown in Fig. 3a and d. To reproduce the environment of the supported MLG on a substrate, hBN was utilized as a substitute for the SiO_2_ substrate. MLG and hBN sheets were obtained by direct mechanical exfoliation onto polydimethylsiloxane (PDMS) stamps. After identifying the number of graphene layers on the PDMS using the optical transmittance difference, hBN and MLG were sequentially transferred from the PDMS stamps to the TEM grid (for details, see Figure S5 in SI) [18]. The atomic periodic structures were analyzed by comparing the selected area electron diffraction (SAED) patterns before and after excimer UV irradiation using the TEM grid holes. For the SLG, the optically distinguishable features of a single layer were identified during the transfer process (see Figure S6 in SI) [19]. The atomic structures were verified by obtaining the SAED patterns for the holes containing SLG and hBN, as shown by the green area in Fig. 3a. After excimer UV irradiation, the completeness of the etching process was confirmed by comparing the SAED patterns. As shown in the innermost part of Fig. 3b, two sets of hexagonal diffraction patterns were observed, maintaining an arbitrary rotation angle. Among them, the brighter and clearer hexagonal diffraction arises from a few-layer hBN, attributed to its greater structural information, whereas the diffraction pattern of the thinner SLG appears relatively faint (see Figure S7 in SI). Further analysis was performed by evaluating the intensity profiles of diffraction patterns. In the SAED patterns, SLG exhibited an intensity ratio below 1 between the first and second diffraction spots, corresponding to {1-100} and {1-210}, respectively, as labeled by Bravais–Miller indices [20]. The signal from the SAED pattern of SLG in Fig. 3b, which had an intensity ratio of 1:1, disappeared after 240 s of UV irradiation. Moreover, only the hBN signal remained, as shown in Fig. 3c, confirming the elimination of SLG [21, 22]. 

Similarly, TEM measurements were performed for the BLG samples both with and without hBN. The intensity profile of the diffraction spots along the rectangular box for BLG only at Hole 1 (Fig. 3d) exhibited an intensity ratio of approximately 1:4, resulting from the AB-stacking nature of BLG (see Figure S8 in SI) [21, 23]. Additionally, TEM measurements were also conducted on Hole 2 of BLG/hBN before and after excimer UV irradiation, and the SAED patterns with intensity profiles are shown in Fig. 3e and f. The subtle discrepancy between the ratios of the {1-100} and {1-210} peaks before and after UV irradiation was attributed to double scattering caused by hBN and the relative lack of structural information owing to its small thickness compared to that of hBN. (see Figure S9 in SI). However, the negligible changes in the SAED signals of BLG on hBN after UV irradiation indicate that BLG remained undamaged by excimer UV, unlike SLG. This supports the effectiveness of the excimer UV etching process in selectively eliminating SLG, while leaving MLG/hBN unaffected. This implies that our selective SLG etching method is suitable for 2D heterostructures, particularly those containing hBN, which is commonly used and has attracted considerable attention for controlling the graphene environment [24–26]. Additionally, these results were supplemented by AFM stick-slip imaging using friction measurements in the contact mode. Stick-slip measurements sense surface atoms only under the conditions of perfect periods of atomic arrangement and clean surfaces. By verifying the preserved hexagonally structured atomic surface of FLG after excimer UV exposure, we showed that its surface was intact at the atomic level (see Figure S10 in SI).

### Facile fabrication of 1D electrode contacts using SLG selective etching

**Fig. 4:**
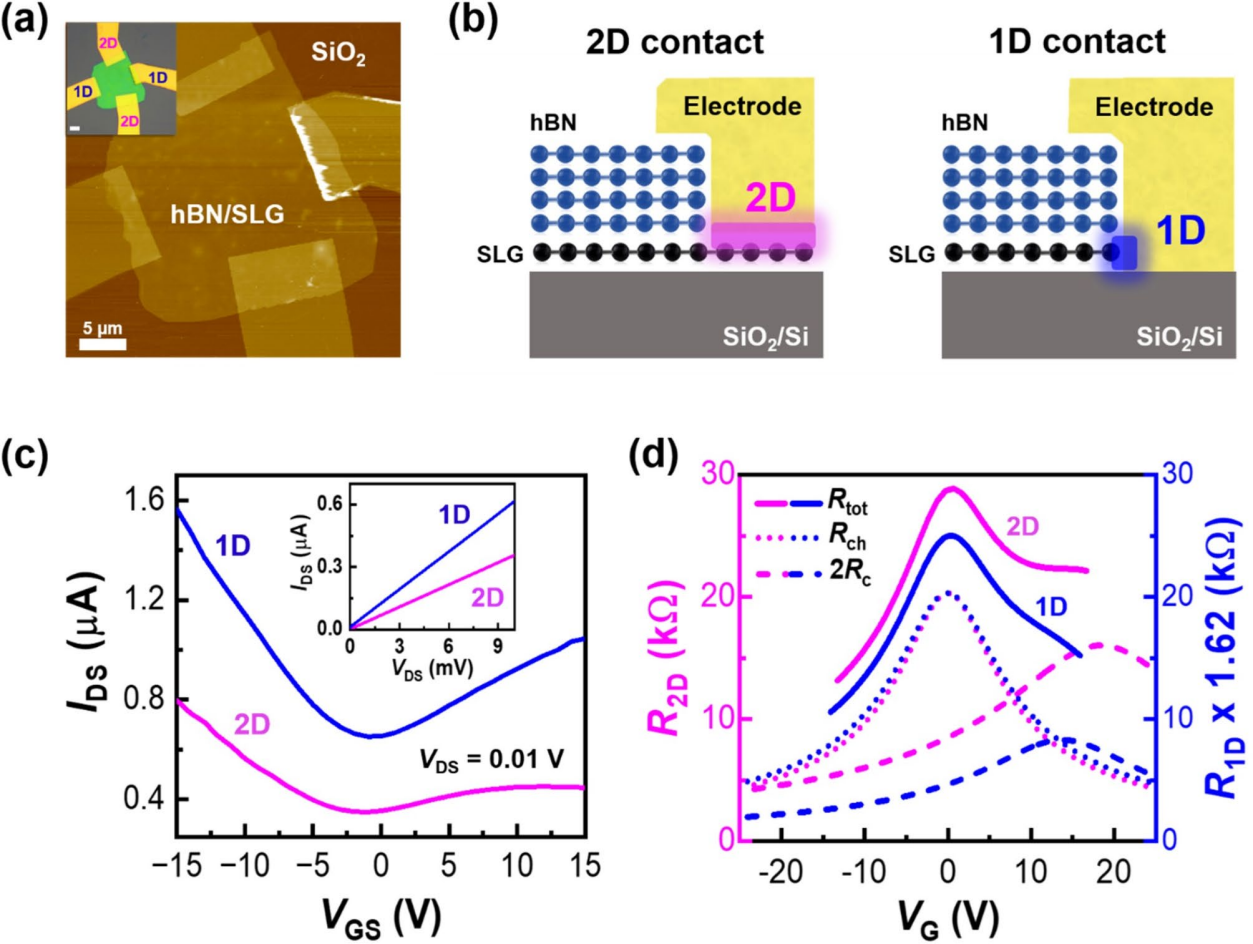
Fabrication of 2D and 1D electrode contacts in an hBN/SLG FET device using SLG selective etching. (**a**) AFM results of the hBN/SLG/SiO_2_ device fabrication with 2D and 1D electrode contacts using the SLG selective etching method. The inset shows an optical image indicating 2D and 1D electrode contacts. (**b**) Schematics of cross-sectional structures of 2D and 1D electrode contact elements. The 2D and 1D contact interfaces between graphene and Au/Cr electrodes are highlighted with magenta and blue colors, respectively. (**c**) Gate-dependent electrical transport characteristics of the 2D and 1D electrode contacts in the hBN/SLG FET device. The inset shows output curves through the drain source. (**d**) Fitting results by the Drude model using the gate-voltage (V_G_)-dependent resistance of 2D and 1D contacts obtained from (**c**). All resistance values of R_tot_, R_ch_, and 2R_C_ in 1D contact are multiplied by 1.62

From an application perspective of selective etching, the most urgent requirement is the fabrication of edge contacts (1D contacts) between 2D materials and metal electrodes. Because 2D materials lack surface bonding sites, connecting a metal electrode via surface contacts (2D contacts) results in strong orbital hybridization, leading to a high contact resistance (*R*_C_) [27, 28]. In contrast, 1D contacts exhibit a lower *R*_C_ than 2D contacts because of the abundance of dangling bonds at the cut edge, which form significantly shorter covalent and ionic bonds between the metal and carbon atoms [29, 30]. Utilizing the features of selective etching via an excimer UV lamp, we designed both 2D- and 1D-contacted electrodes in an hBN/SLG heterostructure sheet. By introducing excimer UV etching, the FET device fabrication procedure was significantly simplified compared with the conventional 1D-contacted SLG FET device. The hBN/SLG was prepared by transferring mechanically exfoliated thin hBN flakes (thickness ≈ 38 nm) onto CVD-grown SLG/SiO_2_ using the PDMS stamping method. The 2D-contacted electrode was directly formed using e-beam lithography and an e-beam evaporator without etching process. Subsequently, the 1D-contacted electrode was sequentially formed after the selective etching of SLG through excimer UV irradiation for 240 s under ambient conditions (see fabrication details in Figure S11 in SI). The resulting AFM topographic and optical microscopic images are presented in Fig. 4a, where the hBN/SLG sheet on the SiO_2_ substrate with a clear-cut edge is shown. The role of the hBN nanosheets as a photomask was confirmed by AFM, Raman spectroscopy, and SEM (see Figure S12 in SI). Schematics of the 2D and 1D contact structures are presented in Fig. 4b.

Figure 4c presents the current–gate voltage curves of the FET characteristics measured for the 2D- and 1D-contacted electrodes through a common SLG channel underlying hBN. As shown in the inset, the *I*_DS_–*V*_DS_ curves show typical ohmic contact behaviors for both the 2D and 1D contacts. A notable difference between the two curves is the current level, corresponding to the total resistance (*R*_tot_) difference owing to the difference in 2*R*_C_. Comparing the FET transport characteristics between the 2D and 1D contacts, both the channel resistance (*R*_ch_) and *R*_C_ exhibited gate-field-dependent Dirac behaviors [31]. The transmission line method and four-point probe measurements were used to extract the separate Dirac behavior of *R*_ch_ and *R*_C_. In our case, the shape of the curve exhibited asymmetrical characteristics around the y-axis, likely influenced by two Dirac points located near 0 V and in the positive voltage region. To confirm this, fitting of the *R*-*V*_G_ curve was performed using the Drude model, considering two Dirac points, and the results were summarized as *R*_tot_=*R*_ch_+2*R*_C_ (see Figure S13 in SI). Figure 4d shows the fitting results with a simplified relationship between *R*_tot_, *R*_ch_, and 2*R*_C_. To compare only the effect of 2*R*_C_ on the 2D and 1D contacts, *R*_ch_ was standardized to the same value using simple multiplication. Interestingly, the *R*_ch_ curves of the 2D and 1D contacts matched in terms of curvature and displacement by a simple multiple of 1.62 times. As a result, the common effect of *R*_ch_ may be omitted, and only the relative 2*R*_C_ effects between the 2D and 1D contacts may be compared. Although the exact area occupied by 2*R*_C_ cannot be specified, the same trend was observed as depicted in the gate dependence of *R*_ch_ and *R*_C_ through four-point probe measurements [30]. It was confirmed that the electrical characteristics of 2D and 1D differed owing to the difference in the relative 2*R*_C_. We summarized the *R*_ch_, *µ*_ch_, *n*_ch_, and 2*R*_C_max_ of 2D and 1D contacts in Table 1. The 1D-contacted device demonstrated superior performance in terms of the charge transport characteristics of the SLG FET compared to the 2D-contacted device through repeated experiments (see Figure S14 in SI). This supports the improvement of electrical transport characteristics by reducing *R*_C_ through contact geometry in an hBN/SLG channel FET device.

**Table 1:** Electrical transport parameters extracted from Drude model fitting calculations

	2D Contact	1D Contact
*R*_ch_max_(kΩ)	20.30	12.52
*µ*_ch_(cm^2^/V·s)	1853.5	2479.4
*n*_ch_(10^11^cm^-2^)	3.9	4.3
2*R*_C_max_(kΩ)	16.02	5.11

## Conclusion

We demonstrated the effectiveness of excimer UV irradiation for the selective etching of SLG from MLG structures. The etching selectivity of SLG was confirmed through comprehensive analyses, including optical microscopy, AFM, Raman spectroscopy, and TEM measurements. Importantly, our simplified approach of 1D edge-contact FET device fabrication using selective etching of SLG yielded significantly enhanced electrical transport characteristics compared to conventional 2D contacts. Moreover, this novel approach has the advantages of high precision, effectiveness, and cleanliness, which are crucial for realizing the full potential of graphene for next-generation 2D electronics. Notably, this approach significantly streamlines device manufacturing by integrating cleaning operations into the etching process, addressing various issues related to contamination. Moreover, the proposed approach ensures cleaner and more efficient manufacturing processes for diverse applications in the 2D electronics industry. This study also provides valuable insights into the tailoring of SLG structures for diverse applications.

## Methods/Experimental

### Characterization and measurements

#### Excimer UV etching

SLG etching was conducted using a 172 nm excimer UV lamp with an illumination power of 11.2 mW/cm^2^ under ambient conditions. The etching parameters were optimized using Raman spectroscopy and TEM.

#### AFM topography

The surface morphology and thickness of the samples were examined using non-contact AFM (Park Systems Inc. NX10) equipped with a non-contact atomic force microscope tip (Nanosensors, NCHR).

#### Raman spectroscopy

Raman spectra and mapping images were obtained using a Raman Spectroscopy System (WITEC UHTS 300) at the Core Facility Center for Quantum Characterization/Analysis of Two-Dimensional Materials and Heterostructures. Measurements were performed at room temperature with a 532 nm laser source (2.33 eV) and a ×100 objective lens. The Raman spectra were normalized to the Si peak (520 cm^-1^) intensity on the SiO_2_ substrate.

*TEM measurement*: Graphene and hBN flakes (purchased from HQ Graphene) were mechanically exfoliated from the bulk form onto the PDMS substrates using a 3M Scotch tape. The dry transfer of hBN was first executed onto a SiN_x_ TEM grid containing holes (Norcada, P/N: NH050D2). Subsequently, graphene was transferred onto the hBN on the TEM grid. TEM measurement was performed at 200 kV with an electron dose rate of approximately 2.0 × 10^6^ e·nm^-2^·s^-1^ (JEOL 2100 F) for prepared SLG/hBN, BLG/hBN, and MLG/hBN stacks.

### Fabrication of graphene FET device

#### Sample preparation

The graphene channels of the FETs were supported on SiO_2_/Si substrates, where p^+^-doped Si was used as the bottom-gate electrode and SiO_2_ (300 nm thermal oxide) as the gate dielectric. The Si/SiO_2_ substrates were cleaned by sonication in acetone, isopropanol, and ethanol and subsequently rinsed with deionized water. SLG was obtained from single-crystalline graphite bulk (HQ graphene) by mechanical exfoliation using an adhesive tape (Scotch tape, 3M) and verified using optical microscopy (see Figure S6 in SI). In addition, the hBN/SLG stacks were prepared using CVD graphene and mechanically exfoliated hBN, employing the dry transfer method with a PDMS stamp and the dry transfer system of a low-profile ball bearing stage (Newport Inc.).

#### Device fabrication

Graphene FET devices were fabricated on SiO_2_/Si substrates via a lithographic process with a spin-coated thin film of polymer poly (methyl methacrylate) (PMMA) resist using e-beam lithography (field emission scanning electron microscope, TESCAN) and an e-beam evaporator (Korea vacuum tech. KVE-E2000). Cr (10 nm) and Au (35 nm) metal electrodes were deposited as the source and drain, respectively. In FET devices containing the SLG channel covered with hBN, 1D and 2D electrode contacts were formed. First, 2D contacts between the CVD–SLG and electrodes were established. Subsequently, after excimer UV etching of the SLG, electrodes for the 1D contact were deposited on the edge side of hBN/SLG (see Figure S11 in SI).

#### Electrical transport measurement

The electrical transport properties of SLG-based FET devices were measured in a vacuum chamber with a high vacuum level of ∼ 1.0 **×** 10^− 6^ mTorr using a vacuum probe station, semiconductor characterization system (MS tech, M6VC), and parameter analyzer (Keithley 4200). The measurements were performed with two-probe contacts to the source and drain to obtain *I*–*V* curves and gate-voltage-dependent electrical transport curves.

### Electronic supplementary material

Below is the link to the electronic supplementary material.

Supplementary Material 1

## Data Availability

The data that support the fndings of this study are available from the corresponding author upon reasonable request.
