# Supplementary material for: Excimer-ultraviolet-lamp-assisted selective etching of single-layer graphene and its application in edge-contact devices
Source: Nano Converg. 2024 Aug 22;11:34. doi: 10.1186/s40580-024-00442-5 (PMC11341517; doi:10.1186/s40580-024-00442-5)
Supplement: Supplementary file 1 — Supplementary Material 1 [file 40580_2024_442_MOESM1_ESM.docx]

Supporting Information

Excimer-ultraviolet-lamp-assisted selective etching of single-layer graphene and its application in edge-contact devices

*Minjeong Shin^1†^, Jin Hong Kim^1†^, Jin-Yong Ko^1^,* *Mohd Musaib Haidari^1^, Dong Jin Jang^1^, Kihyun Lee^2^, Kwanpyo Kim^2^, Hakseong Kim^3^, Bae Ho Park^1*^, Jin Sik Choi^1*^*

^1^Department of Physics/Division of Quantum Phases and Devices, Konkuk University, 120 Neungdong-ro, Gwangjin-gu, 05029, Seoul, Republic of Korea
^2^Department of Physics, Yonsei University, 50 Yonsei-ro, Seodaemun-gu, 03722, Seoul, Republic of Korea

^3^Korea Research Institute of Standards and Science (KRISS), 267 Gajeong-ro, Yuseong-gu, Daejeon 34113, South Korea

^*^Corresponding authors. E-mail: [jinschoi@konkuk.ac.kr](mailto:jinschoi@konkuk.ac.kr), [baehpark@konkuk.ac.kr](mailto:baehpark@konkuk.ac.kr)

**Table S1.** Comparison of various UV-based etching techniques for graphene patterning.

| **UV Source** | **Etching effect** | **Environment** | **Power** | **Treatment time (min)** | **Reference** |
| --- | --- | --- | --- | --- | --- |
| UV  (Hg-Xe) | Wet etching  using mask | NaClO etchant | 3.5 W/cm^2^ | 5 | [1] |
| UV (Hg-Xe) | Etching edge of graphene | Ambient with Applied external electric field | 1.0 W/cm^2^ | 20 | [2] |
| Excimer UV (Xe_2_) | H_2_O-based photochemical etching using mask | Low-pressure H_2_O atmosphere (20 Pa) under magnetic field | ~20 kW | 1 | [3] |
| Excimer UV (Xe_2_) | N_2_-based photochemical etching using mask | Low-pressure N_2_ atmosphere (10 Pa) under magnetic field | ~1.5 kW | 40 | [4] |
| Excimer UV (Xe_2_) | Selective single-layer graphene etching | Ambient | 11.2 mW/cm^2^ | 4 | This work |


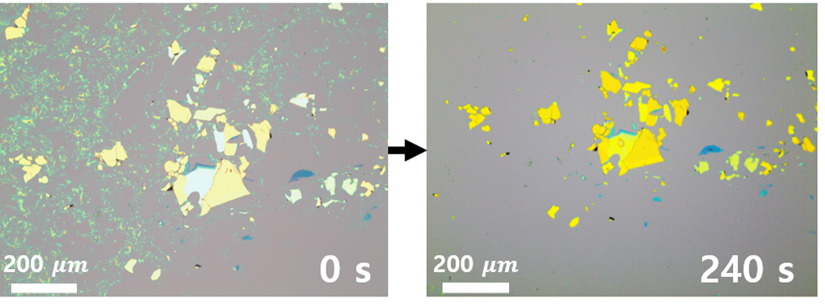


**Figure S1**. Cleaning effect of graphene sample on SiO_2_/Si substrate by excimer UV treatment. Polymeric sticky tape residue was removed after excimer UV irradiation for 240 s.


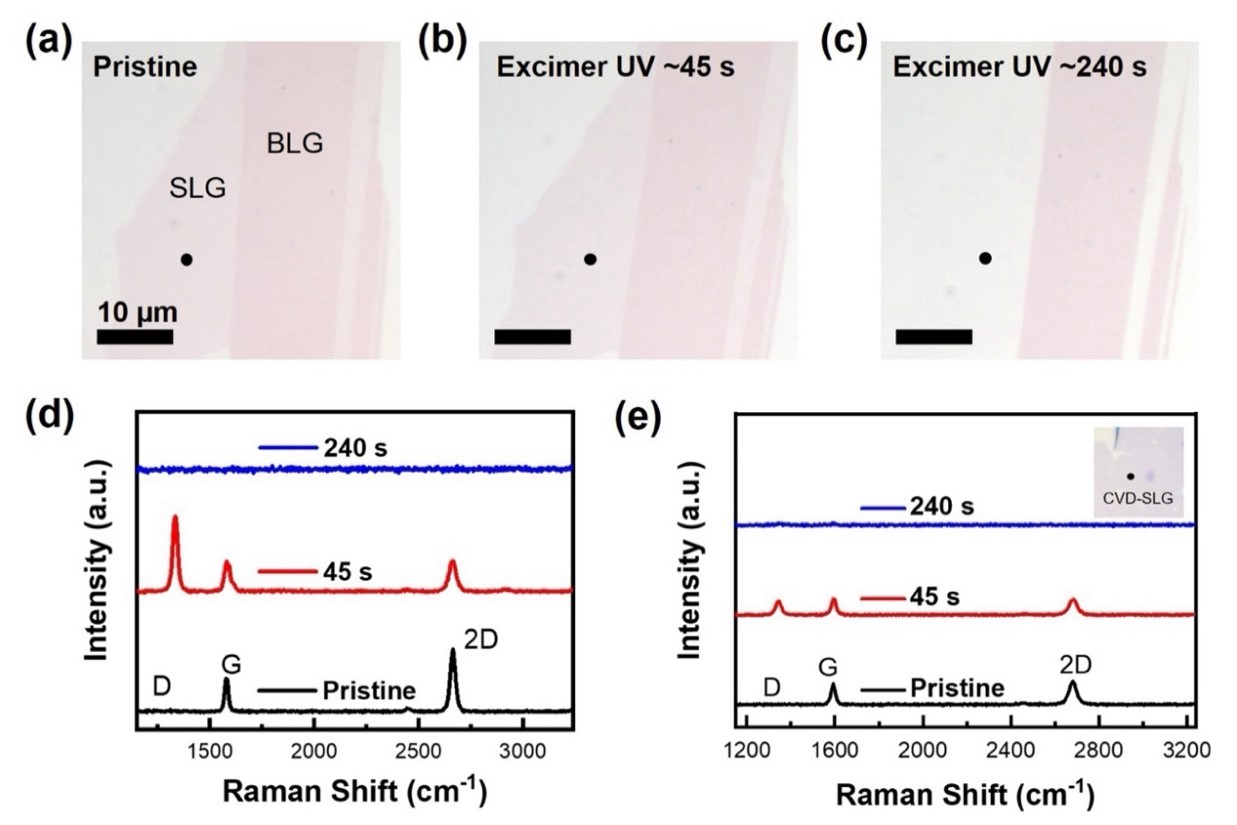


**Figure S2**. Optical microscopy images of mechanically exfoliated graphene and Raman spectroscopy. (a) – (c) Optical microscopy images of mechanically exfoliated graphene on SiO_2_/Si substrate after different exposure durations of excimer UV irradiation. The irradiation time is designated in each figure. (d) Raman spectroscopy obtained at the SLG region designated as black dots in (a) - (c). (e) Raman spectrum changes for CVD-grown SLG under identical excimer UV irradiation conditions, exhibiting comparable results to the mechanically exfoliated SLG. The inset exhibits the measurement location on CVD-SLG.


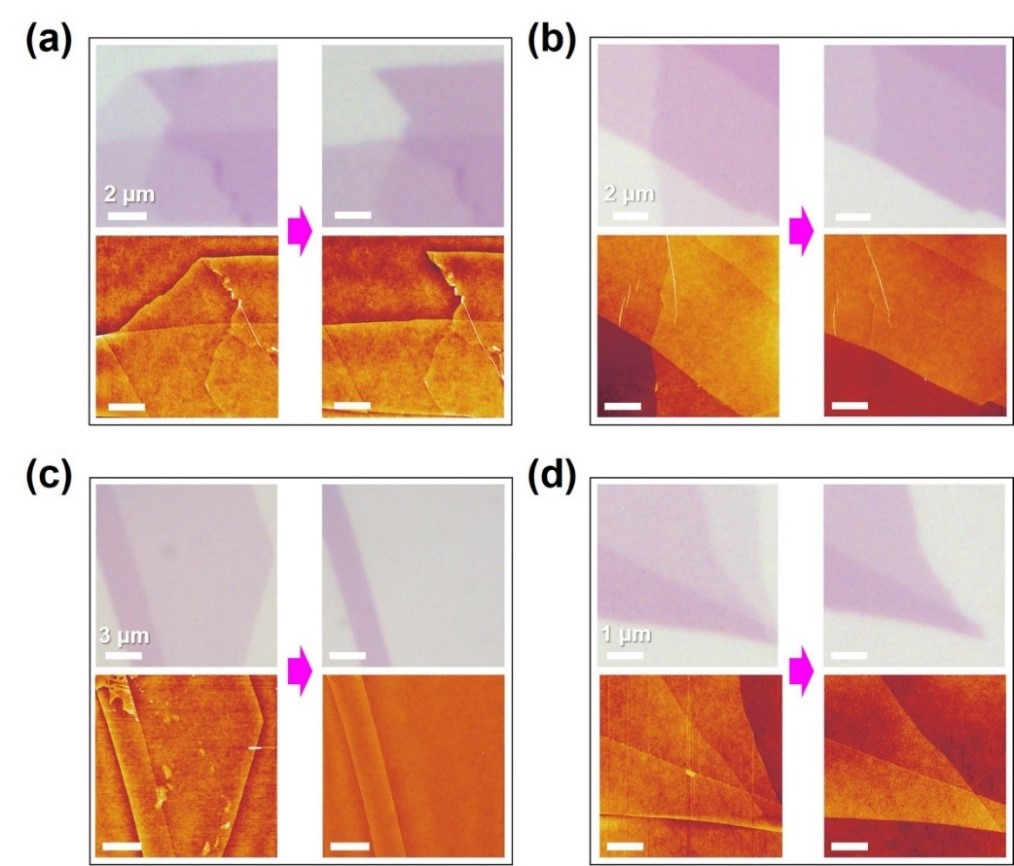


**Figure S3**. (a) – (d) Sets of optical microscopy and AFM topography images of multilayered graphene samples before and after excimer UV irradiation, showing the selective etching of SLG. The AFM topography images before and after excimer UV treatment not only reveal detailed edges and structures remaining intact, such as rippled edges and wrinkles but also clearly show that raised edges have subsided and residue has been removed.


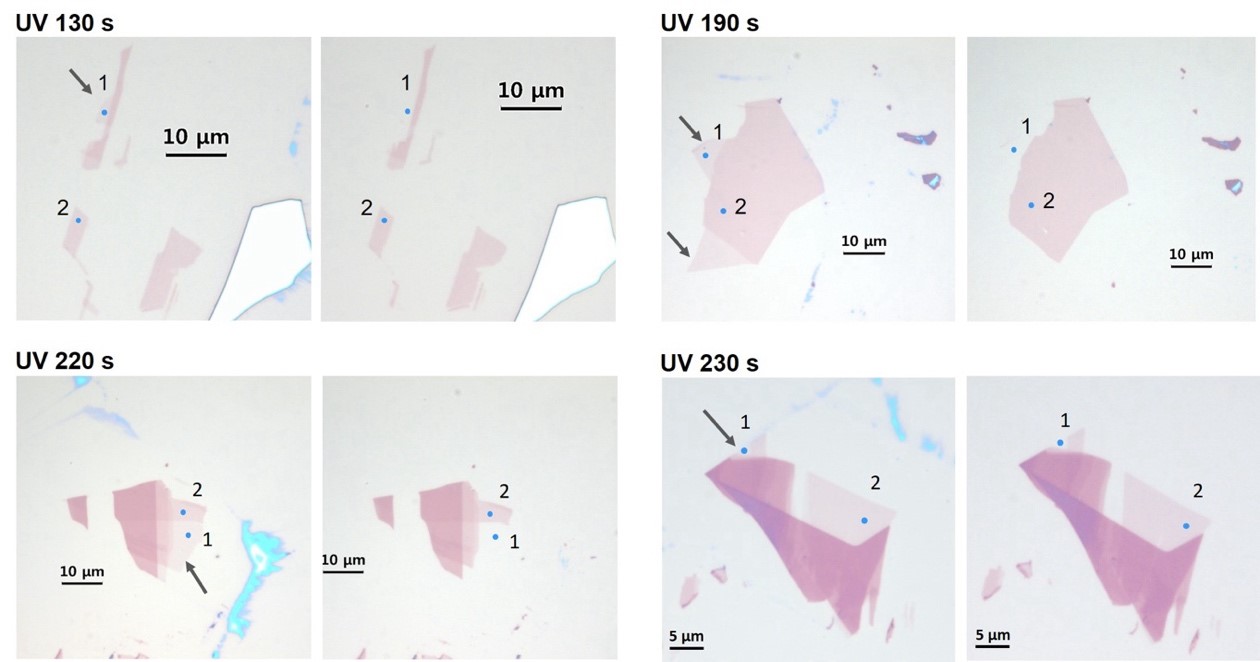


**Figure S4**. Optical images of graphene samples captured before and after excimer UV exposure ranging from 130 s to 230 s. The blue dots marked with numbers represent SLG (1) and BLG (2), indicating the respective positions for Raman spectroscopy measurements.

  
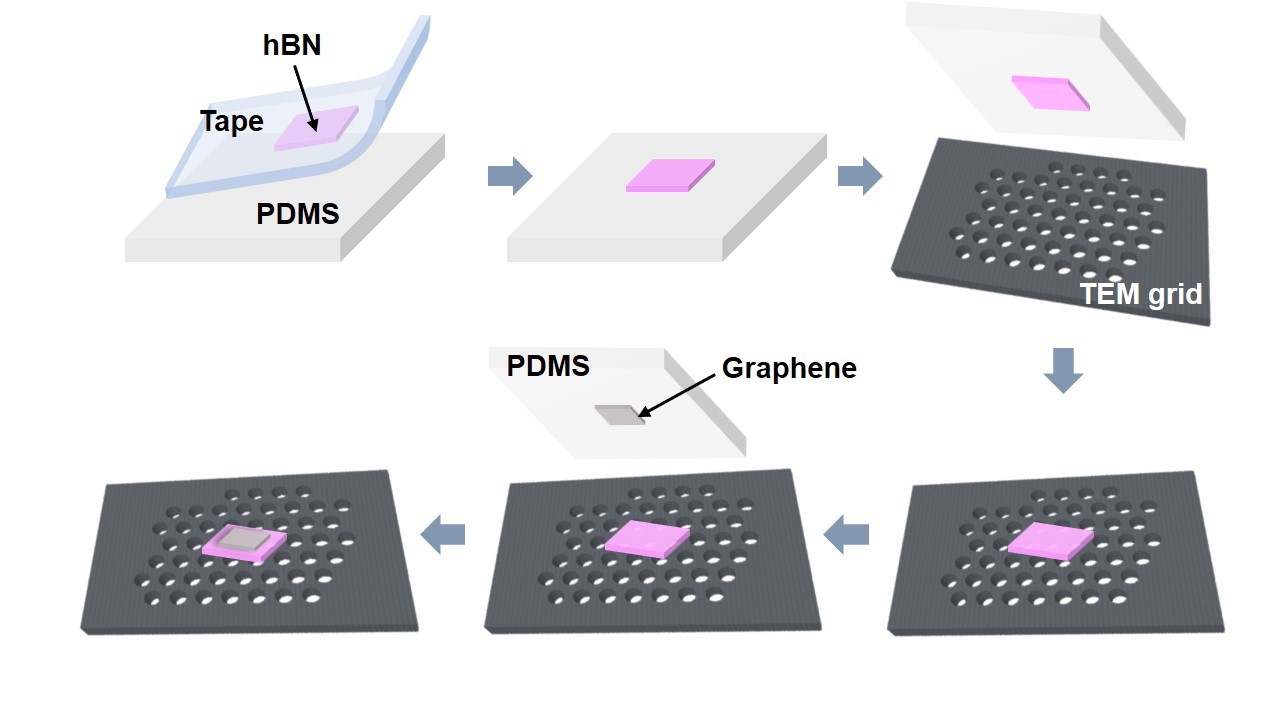


**Figure S5**. Schematic of the sample preparation process for TEM measurement. An hBN flake was obtained by using 3M tape on a PDMS stamp and then dry-transferred to a TEM grid. Similarly, to fabricate the graphene/hBN sample, graphene was also transferred from PDMS onto the hBN/TEM grid.


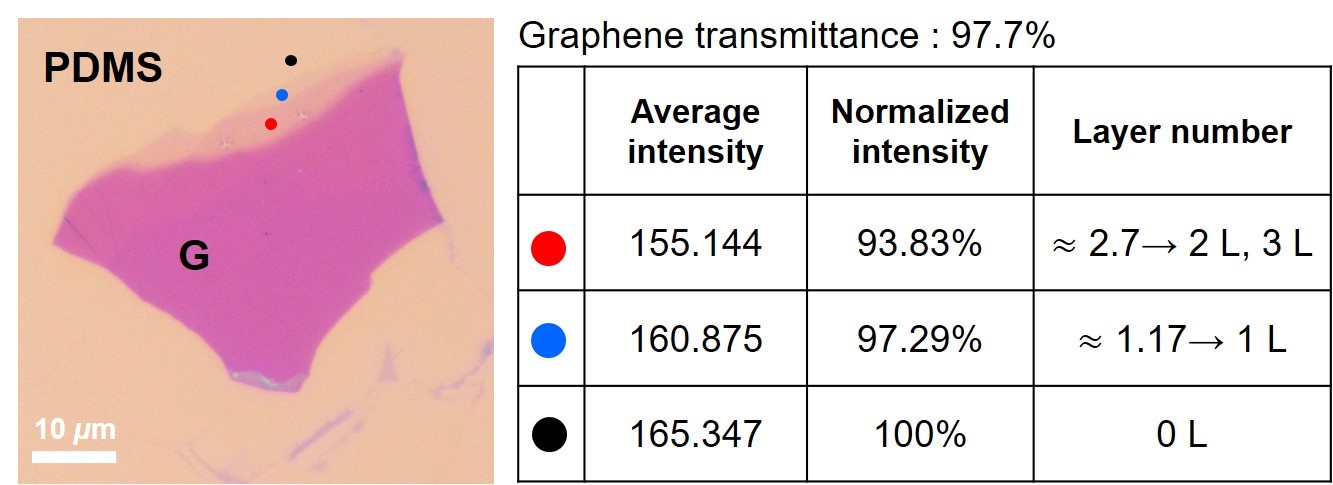


**Figure S6**. Determination of the number of graphene layers on PDMS by analyzing the difference in optical transmittance before transferring onto hBN. Black, blue, and red dots present areas of PDMS without graphene, SLG, and few-layer graphene, respectively. Utilizing the transmittance of SLG at 97.7%, the number of graphene layers was estimated from the normalized light intensity.^[5]^


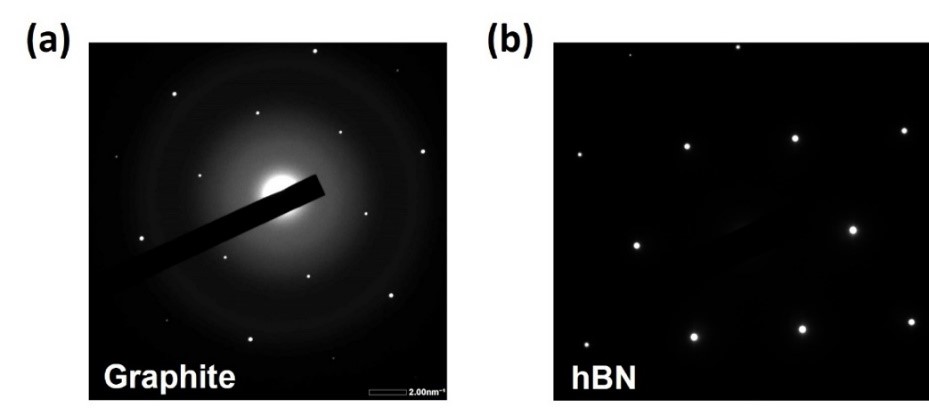


**Figure S7**. SAED patterns of (a) graphite and (b) hBN obtained from TEM measurements.


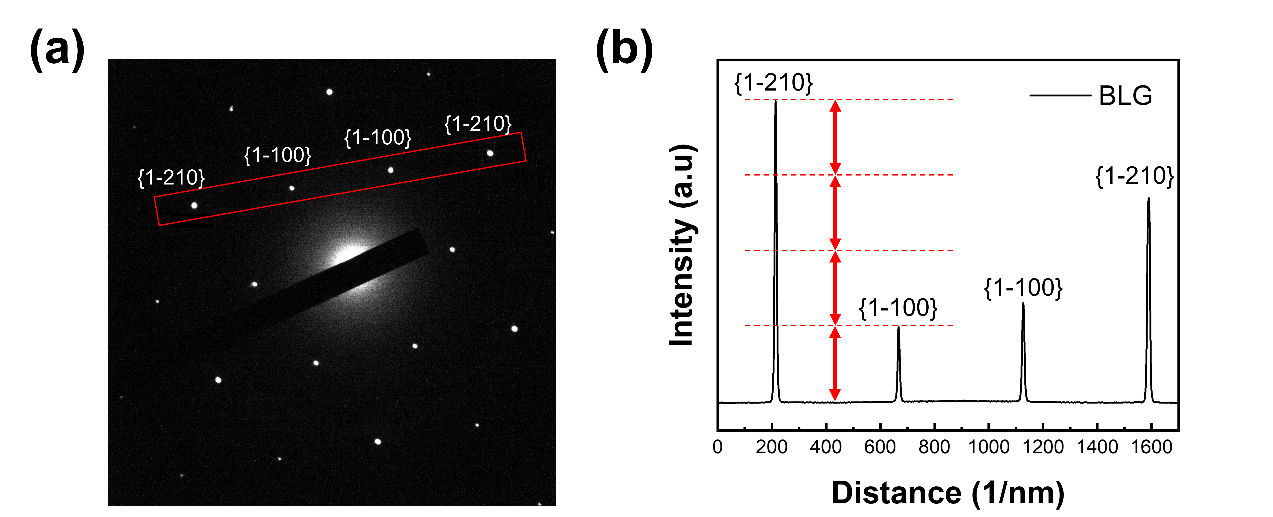


**Figure S8**. (a) SAED pattern image obtained from TEM measurement for BLG on the TEM grid. (b) Intensity profile plot of the region highlighted by red box in (a). Red arrows and dashed lines indicate the intensity ratio of 1:4 between {1-100} and {1-210}.


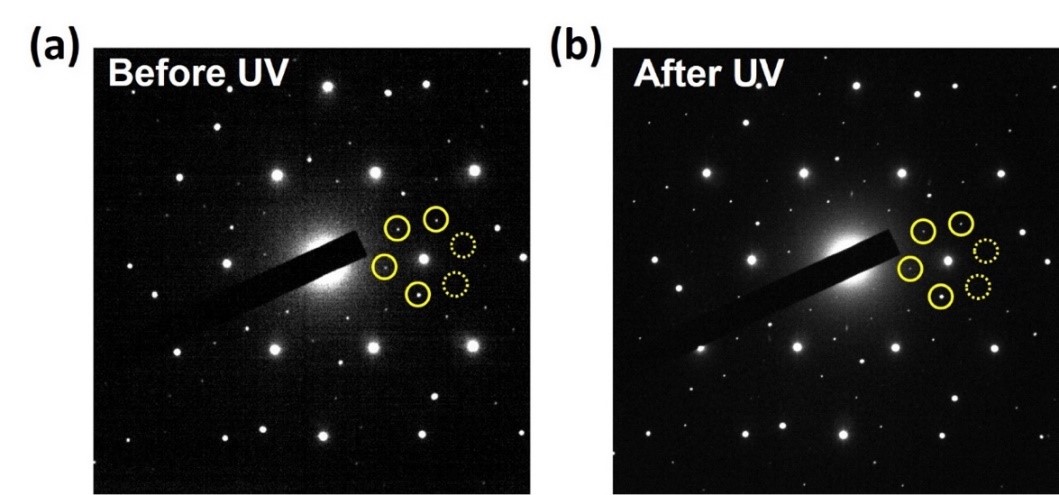


**Figure S9.** SAED patterns of TEM measurements (a) before and (b) after excimer UV treatment of 240 s for BLG/hBN. Yellow circles indicate the signals from a double scattering of electrons by a thick hBN flake below BLG, while yellow dashed circles represent the positions where double scattering is expected to result in a pattern with a hexagonal arrangement centered on the hBN peak. Due to this double scattering phenomenon, one of the characteristics of BLG, the 1:4 intensity ratio between {1-100} and {1-210}, is not maintained.


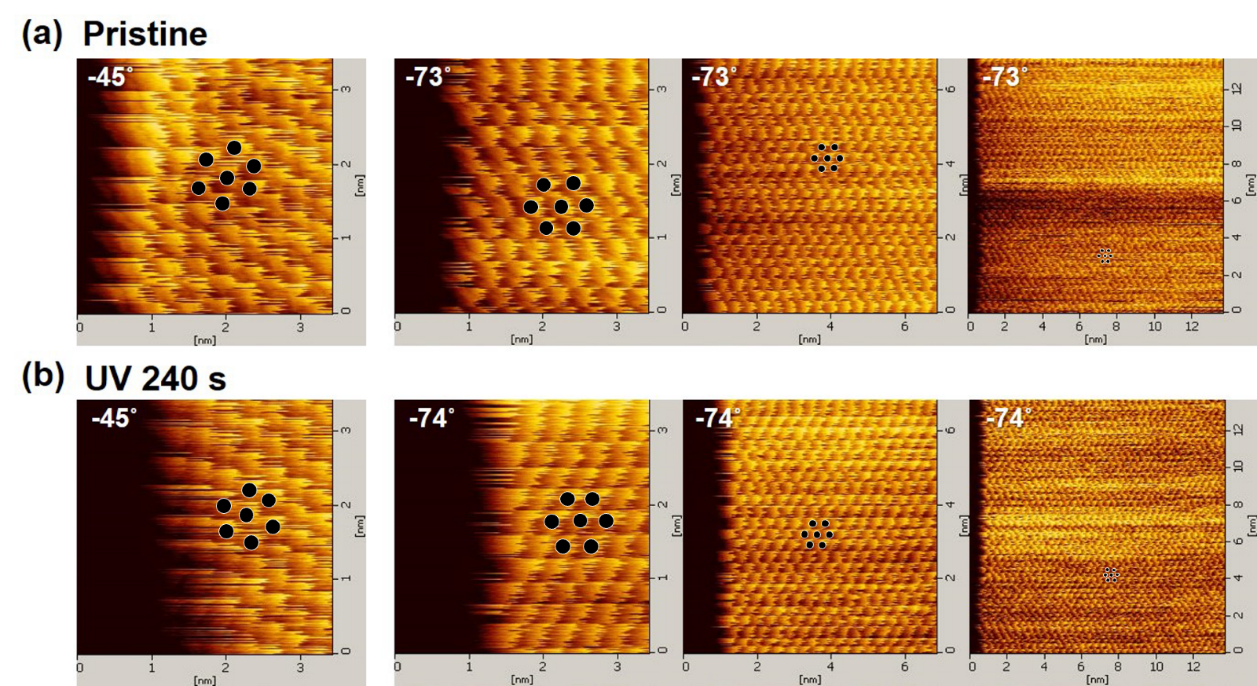


**Figure S10**. AFM stick-slip images on mechanically exfoliated multilayer graphene before and after excimer UV irradiation. The scan lengths are 3.4 nm, 3.4 nm, 6.8 nm, and 13.7 nm from left to right. The scan direction is indicated in the upper left. Representative periodicity is indicated by a set of black dots. Periodic behavior was confirmed in each sample through changes in scan direction and scan area.


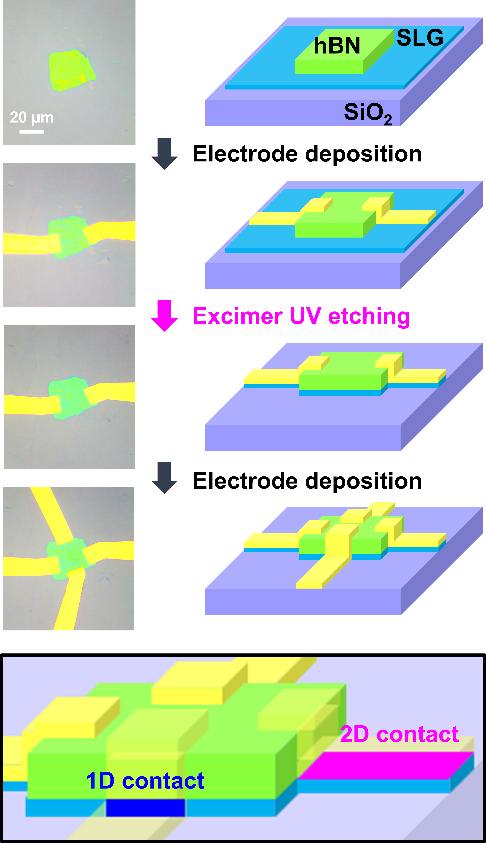


**Figure S11**. Schematics and optical images of the fabrication process for a FET device with 1D and 2D contacts. Dry transferring hBN was performed onto CVD-SLG on SiO_2_/Si substrate, followed by the deposition of Cr and Au to form 2D contacts between the metal electrodes and graphene. After excimer UV etching, the graphene area exposed under the electrodes and hBN remained unetched. Finally, Cr/Au electrodes were deposited on the side of hBN/SLG for 1D contact.


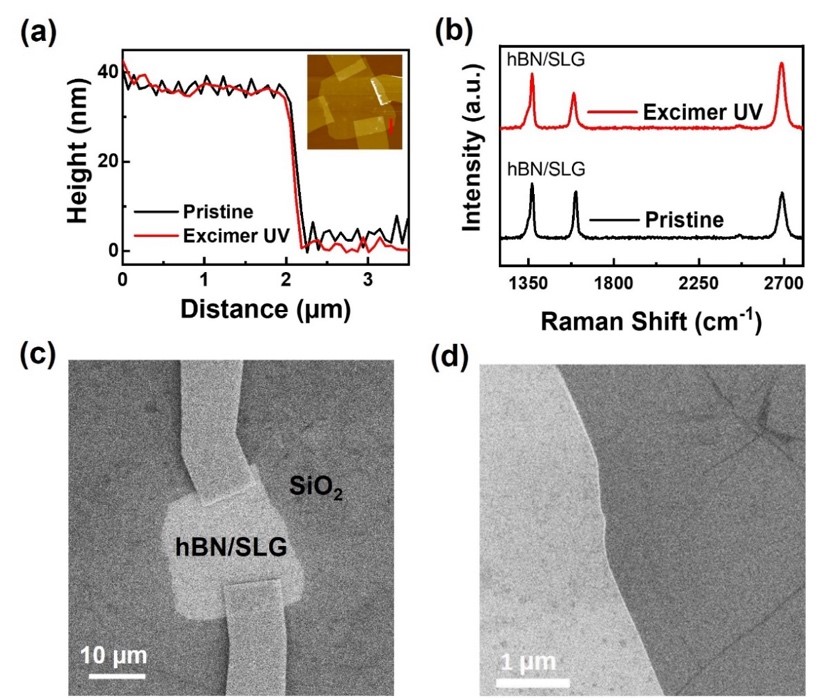


**Figure S12**. AFM and SEM measurements provide high-resolution images of material surfaces. (a) Line profile extracted from AFM topography of hBN/SLG before and after excimer UV irradiation. The difference in height between them shows decreased thickness due to the removed SLG area. (b) Raman spectroscopy of hBN/SLG before and after excimer UV irradiation. The result shows no noticeable change, implying that SLG covered by hBN is protected from the impact of excimer UV. (c) SEM image of FET device based on hBN/SLG with 2D contacted electrodes after excimer UV irradiation, illustrating its clear and sharp cut edge. (d) Enlarged SEM image of the hBN/SLG edge area in (c).

AFM and SEM measurements provide high-resolution images of material surfaces. Figure S12a demonstrates a significant increase in the height difference between hBN and SLG after excimer UV irradiation. Conversely, SLG regions covered by hBN exhibit negligible height changes upon excimer UV irradiation. Raman spectroscopy of excimer UV-irradiated hBN/SLG further reveals that hBN effectively preserves the underlying SLG structure, as shown in Figure S12b. Additionally, SEM images of the hBN/SLG heterostructure following excimer UV etching (Figure S12c and d) exhibit a distinct etched cut edge.


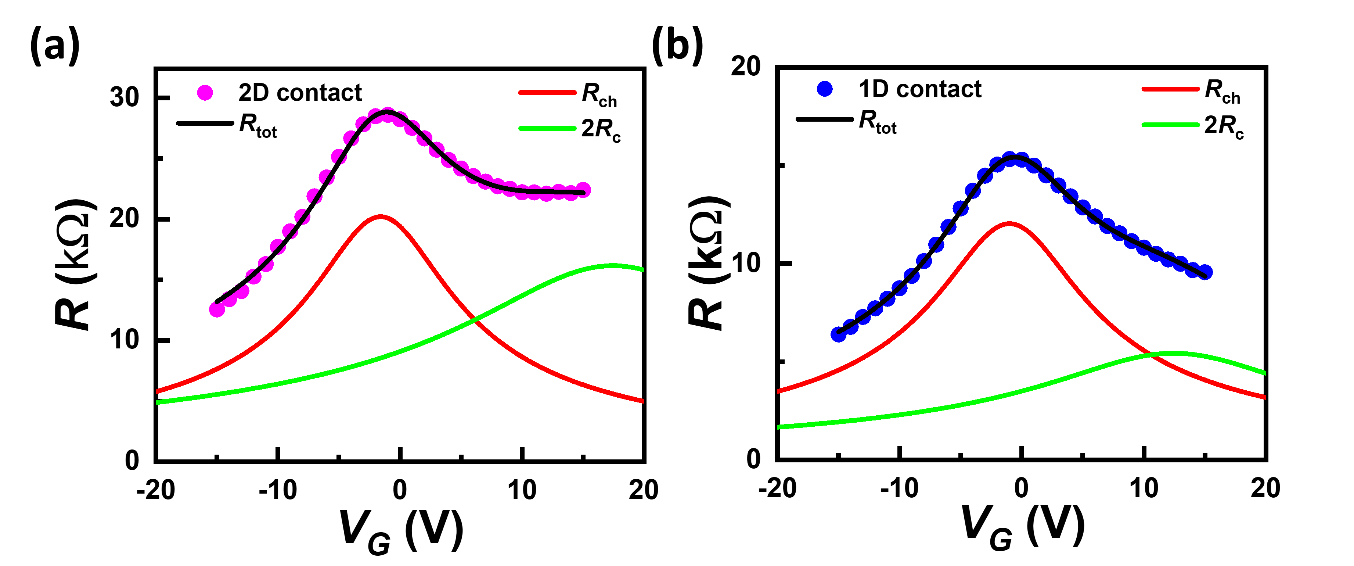


**Figure S13**. Comparison of electrical transport properties obtained through (a) 2D contacted, and (b) 1D contacted electrodes for the common hBN/SLG FET. The values of *R*_ch_ and 2*R*_C_ were extracted through fitting using the Drude model.

  Assessment of electrical transport characteristics in graphene FETs commonly utilizes the Drude model fitting method^[6]^:

$$R_{tot}={2R}_{C}+\left( \frac{L/W}{q\mu\sqrt{\left( \frac{C_{ox}\left( V_{G} - V_{D} \right)}{q} \right)^{2}+n^{2}}} \right)$$

where *L* and *W* represent the length and width of the graphene channel, *q* is the electric charge, *V*_G_ is the applied back-gate voltage, *V*_D_ is the Dirac point voltage, *n* is the residual carrier concentration, and *C*_ox_ is the gate capacitance of SiO_2_. In this fitting procedure, two Dirac curves of *R*_ch_ and 2*R*_C_ are considered, confirming the coincidence of the *R*_ch_ curves extracted from 2D and 1D contacts by a simple multiplication of 1.62. Figure S13 demonstrates the experimental gate voltage-dependent resistance curves for 2D and 1D contacts with fitting results of *R*_tot_, *R*_ch_, and 2*R*_C_. All parameters are detailed in Table S2. Moreover, electron/hole mobilities were also calculated using the transconductance method expressed as

$$\mu=\frac{L}{WC_{ox}V_{DS}}\left( g_{m} \right)$$

where, *V*_DS_ and *g*_m_ $\left( g_{m}={\Delta I_{\mathrm{DS}}}/{{\Delta V}_{\mathrm{GS}}} \right)$ represent drain-source voltage and transconductance (slope values in *I-V* curve) of the graphene channel. The calculated electron/hole mobilities are presented in Table S3. This total effect of channel and 2*R*_C_ also exhibits improved electrical transport characteristics in 1D contact compared to 2D contact.

**Table S2**. All parameters, calculated in the fitting with the Drude model.

|  | 2D Contact | 1D Contact |
| --- | --- | --- |
| *L*_ch_ (μm) | 23.5 | 21.3 |
| *W* (μm) | 10 | 10 |
| *V*_D_ (V) | -1.7 | -0.93 |
| *n*_ch_ (10^11^ cm^-2^) | 3.9 | 4.3 |
| *μ*_ch_ (cm^2^V^-1^s^-1^) | 1853.5 | 2479.4 |
| *R*_ch_ at *V*_D_ (kΩ) | 20.30 | 12.52 |
| *2R*_C_max_ (kΩ) | 16.02 | 5.11 |

**Table S3**. The hole/electron mobility values of 2D and 1D contacts calculated by transconductance methods.

|  | 2D Contact | 1D Contact |
| --- | --- | --- |
| Hole mobility (cm^2^V^-1^s^-1^) | 1130 | 2070 |
| Electron mobility (cm^2^V^-1^s^-1^) | 278 | 681 |


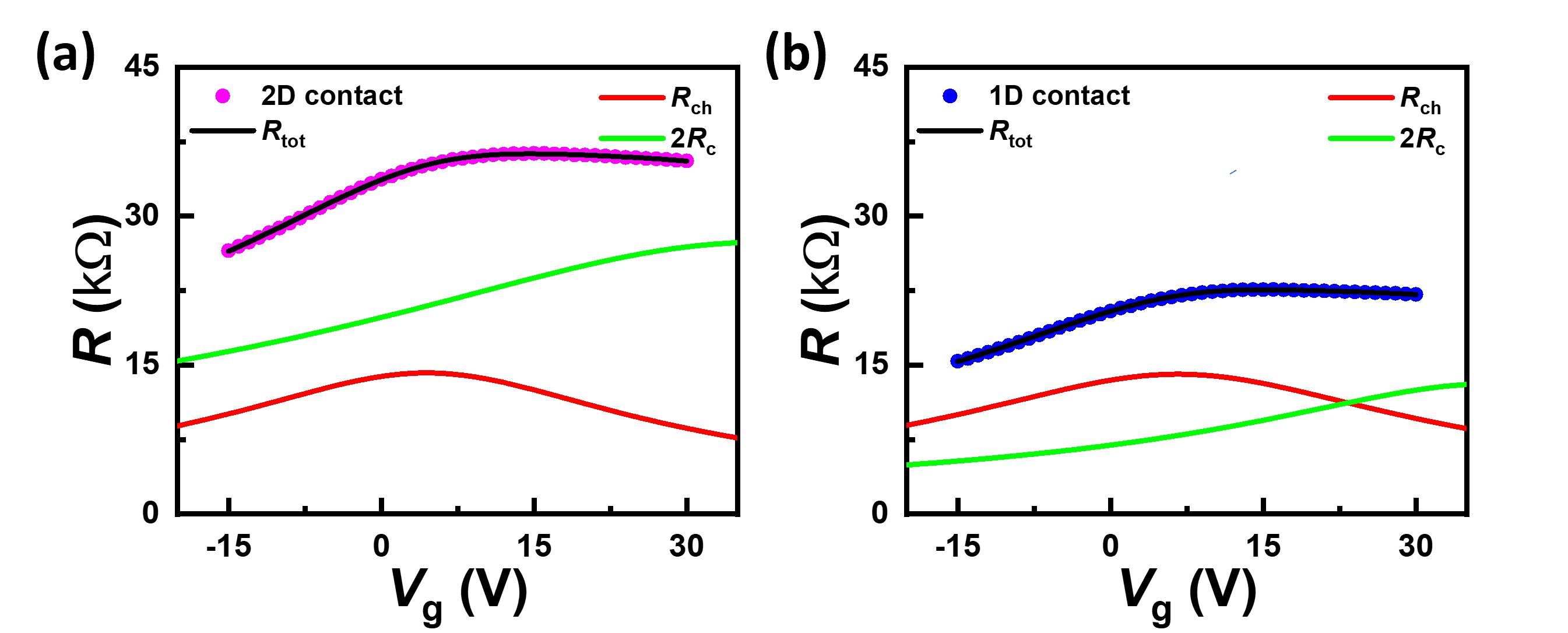


**Figure S14**. Additional electrical transport property set of a hBN/SLG FET device with (a) 2D and (b) 1D contacted electrodes.

References

1. M. Zhang, M. Yang, Y. Okigawa, T. Yamada, H. Nakajima, Y. Iizumi, T. Okazaki. Sci. Rep. **12**, 4541 (2022).

2. N. Mitoma, R. Nouchi. Appl. Phys. Lett. **103**, 201605 (2013).

3. H. Tao, M. Li, Y. Liu, Z. Zhang, F. Wang, Y. Sha, J. Huang, Y. Xu, G. Chen, K. Liu, D. Qian. ACS Appl. Nano Mater. **7**, 10690 (2024).

4. Y. Wu, H. Tao, S. Su, H. Yue, H. Li, Z. Zhang, Z. Ni, X. Chen. Sci. Rep. **7**, 46583 (2017).

5. S. Zhu, S. Yuan, G. C. A. M. Janssen. EPL, **108,** 17007 (2014)

6. H. Zhong, Z. Zhang, H. Xu, C. Qiu, X.-M. Peng. AIP Adv. **5**, 5057136. (2015).
